# Label-free Brillouin endo-microscopy for the quantitative 3D imaging of sub-micrometre biology

**DOI:** 10.1038/s42003-024-06126-4

**Published:** 2024-04-15

**Authors:** Salvatore La Cavera, Veeren M. Chauhan, William Hardiman, Mengting Yao, Rafael Fuentes-Domínguez, Kerry Setchfield, Sidahmed A. Abayzeed, Fernando Pérez-Cota, Richard J. Smith, Matt Clark

**Affiliations:** 1 Optics and Photonics Group, Faculty of Engineering, University of Nottingham, University Park, Nottingham, NG7 2RD UK; 2 Advanced Materials & Healthcare Technologies, School of Pharmacy, University of Nottingham, University Park, Nottingham, NG7 2RD UK

**Keywords:** 3-D reconstruction, Ultrasound, Endoscopy

## Abstract

This report presents an optical fibre-based endo-microscopic imaging tool that simultaneously measures the topographic profile and 3D viscoelastic properties of biological specimens through the phenomenon of time-resolved Brillouin scattering. This uses the intrinsic viscoelasticity of the specimen as a contrast mechanism without fluorescent tags or photoacoustic contrast mechanisms. We demonstrate 2 μm lateral resolution and 320 nm axial resolution for the 3D imaging of biological cells and *Caenorhabditis elegans* larvae. This has enabled the first ever 3D stiffness imaging and characterisation of the *C. elegans* larva cuticle in-situ. A label-free, subcellular resolution, and endoscopic compatible technique that reveals structural biologically-relevant material properties of tissue could pave the way toward in-vivo elasticity-based diagnostics down to the single cell level.

## Abstract (web summary)

A hair-thin fibre-optic imaging system that can image the mechanical properties of single-cellular and multi-cellular organisms in 3D with sufficient resolution to identify 300 nm structures in the C. elegans cuticle.

## Introduction

Exogenous contrast labels are indispensable tools that illuminate invisible microscopic biological landscapes to scientists and clinicians, but also present additional complexities. Several powerful label-free endo-microscopy techniques have emerged over the years in efforts to circumvent the need for tissue labelling, e.g., those based on optical coherence tomography^1^, multiphoton^2^, Raman^3^, and photoacoustic^4^ imaging mechanisms. However, achieving both subcellular resolution in three dimensions and subcellular contrast of biological material properties remains challenging. Brillouin microscopy is an emerging label-free opto-acoustic imaging modality that can potentially meet these challenges and probes the viscoelasticity of tissue undergoing picosecond (*ps*) time-scale deformation and relaxation^5–8^. Brillouin scattering based techniques measure the shift in frequency (Δ*f*_*B*_, Eq. (1) in Methods) of photons that have inelastically scattered with stimulated or spontaneous thermal phonons of frequency *f*_*B*_ (~GHz for biological tissue) and path length ${\alpha }_{B}^{-1}$ where *α*_*B*_ is the phonon attenuation rate^9^. Quantifying these phonon characteristics allows inference of local mechanical information, such as the sound velocity (*v*) and longitudinal modulus (M^*^) provided there is a priori knowledge of the refractive index (*n*) and mass density (*ρ*) (refer to Eq. (2) in Methods for more details).

Advances in spontaneous^10,11^ and stimulated^12^ Brillouin microscopy have unlocked new insights in the mechanobiology of cardiovascular^13^, ophthalmological^14^, neurodegenerative^15^, and bone^16^ diseases, which could drive the technology towards future mechano-histopathology. However, in-vivo endoscopic translation of this technology has been slow, in part due to the large Brillouin frequency shifts stimulated or spontaneously scattered light experiences as it propagates through glass optical fibres. Towards addressing this limitation, recent spontaneous Brillouin spectroscopy techniques have utilised hollow-core fibres^17^ or partially-free-space dual-fibre mechanisms^18^ to demonstrate single-point and 1D spectral acquisitions, yet full 3D imaging has remained elusive.

**Fig. 1:**
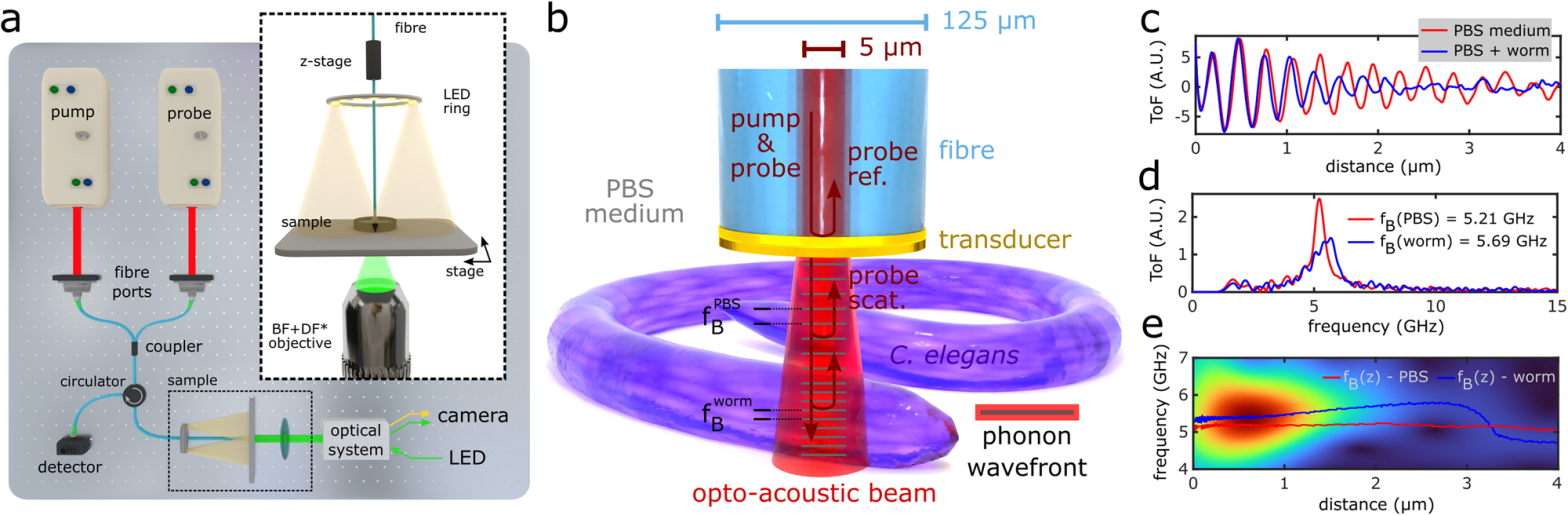
PEM working principle and 3D elastography mechanism. **a** Optical fibre-based PEM system (see Methods), supplemented with brightfield (BF) and darkfield (DF) imaging systems to co-localise optical and phononic measurements. **b** PEM working principle: a fibre-tip transducer optically absorbs a pump pulse which photoacoustically generates ~GHz frequency phonons that propagate longitudinally into the specimen. A series of time-delayed probe pulses reflect (ref.) and transmit through the transducer and Brillouin scatter (scat.) with the acoustic wavefront. Ref. and scat. counter-propagate in the system and interfere at the detector producing a time-resolved signal representing the phonon time-of-flight (ToF). **c** The ToF signals quantify viscoelasticity when analysed in the frequency domain (Fourier transform) as in **d** or through time-frequency analysis as in **e** to obtain depth resolution (red and blue lines represent frequency with maximum amplitude versus *z*).

Here, we present phononic endo-microscopy (PEM) which achieves subcellular resolution in all three spatial dimensions, label-free biomechanical imaging contrast, glass-compatible fibre imaging channels, and simultaneous nano-profilometry. This combination is uniquely enabled by the time-resolved Brillouin scattering (TRBS) process and our optical fibre opto-acoustic transducer technology^19^ (see Fig. 1a, b and Methods). Briefly, the distal tip of the fibre-endoscope photoacoustically emits a longitudinal coherent acoustic phonon (CAP) field with a centre frequency on the order of 100 GHz (see Supplementary Note 1 and Supplementary Fig. 1), into the specimen (Fig. 1b) that is ~6 orders of magnitude greater amplitude than the incoherent spontaneous thermal phonons intrinsic to the glass fibre. This enables an enhanced Brillouin scattering interaction between a near-infrared probe beam (*λ*_*probe*_) and the CAP frequency component that meets the Bragg condition (*λ*_*phonon*_ = *λ*_*probe*_/2*n*).

Instead of measuring the optical Brillouin frequency shift in the frequency domain (e.g., with a spectrometer), the PEM produces a phonon time-of-flight (ToF) signal for which the instantaneous frequency is modulated by depth-resolved changes in the mechanical properties of the specimen^20^ (Fig. 1c–e), i.e., *f*_*B*_(Δ*v*(*z*)). Due to the *ps* temporal sampling rate of the PEM system (see Methods), we are capable of resolving longitudinal changes in viscoelasticity on the order of the phonon wavelength (*λ*_*phonon*_ ~ 300 nm) through time-frequency analysis (TFA) of the ToF signal^20,21^ (Fig. 1e). Therefore, unlike spontaneous and stimulated Brillouin microscopy, the PEM can achieve an unprecedented experimental axial resolution of 320 nm, that is independent of confocal scanning and the 50 μm optical depth of focus of its optical fibre. Optical lateral resolution is dictated by the mode field radius of PEM’s single mode optical fibre and is enabled by point scanning the specimen or PEM. In this work we demonstrate for the first time endo-microscopic 3D elasticity imaging of both single-cellular and complex multi-cellular organisms with 2 × 2 × 0.3 μm ~ 1.2 μm^3^ volumetric resolution and spectrum acquisition speeds of ~ 50 ms/voxel.

## Results

**Fig. 2:**
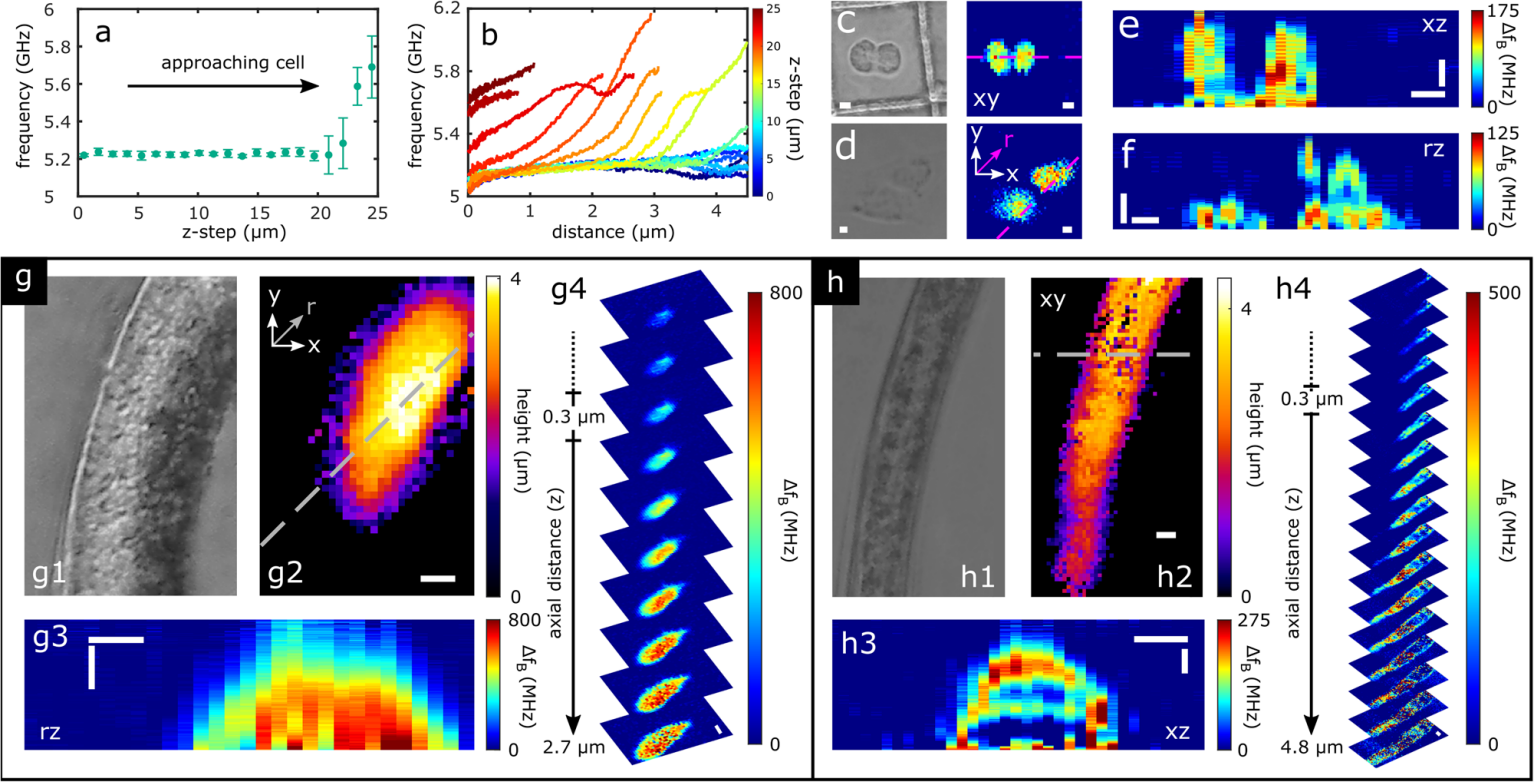
High resolution 3D PEM imaging of single cells and multicellular *C. elegans.* **a** A piezo nanopositioner *z*-steps the PEM fibre-tip to the specimen (HeLa cell) to activate the PEM system (refer to Methods). This is visualised as a change in the weighted average Brillouin shift of the ToF signal (**a**), or as depth-resolved Brillouin shifts (**b**), both as functions of the piezo step. **c** Phase contrast and PEM images of 3T3 fibroblasts; **d** epi-DF and PEM images of MG-63 cells. **e**, **f** Respective cross-sections (along pink lines in **c** and **d** showing subcellular 3D imaging capability. **g** 1-2) epi-DF and PEM profilometry of dehydrated *C. elegans*. **g** 3-4) 3D elasticity imaging (Brillouin frequency shift) of dehydrated *C. elegans* near mid-body reveals a lack of biological structure and high relative stiffness. **h** 1-2) epi-DF and PEM profilometry of hydrated *C. elegans*. **h** 3-4) Comparative 3D imaging of preserved structure of a hydrated *C. elegans*. Horizontal and vertical scale bars are 5 μm and 1 μm, respectively.

Without fluorescent labels, mammalian biological cells in culture typically exhibit maximum intracellular refractive index variation on the order of 0.01 refractive index units (~0.5% relative variation)^22^. Conversely, the intracellular variation of mechanical properties is typically an order of magnitude greater than optical properties. For example, the nucleoli in HeLa cells are 4% stiffer (longitudinal modulus) than the cytoplasm despite only a 0.6% fluctuation in mass density^22^. Adipocytes represent a more extreme example as they can exhibit stiffness variations of ~20% between cytoplasm and lipid droplets^22^. Our previous fibre probes have demonstrated sufficient frequency resolution to observe ~0.3% shifts in stiffness from baseline^19,20^. Taken together with the label-free contrast provided by subcellular mechanics, we use the PEM to 3D image mouse embryonic fibroblast cells (NIH/3T3) and human bone fibroblast cells (MG-63) (both cell lines fixed; see Methods). Contact spectroscopy measurements were initially performed on HeLa cells (Fig. 2a), where the PEM-tip position is controlled with a piezo nanopositioner until a shift in Brillouin frequency relative to the medium-baseline is observed (implying approach and/or contact). However, when TFA accompanies this procedure (Fig. 2b), the frequency boundary of the cell becomes depth-resolvable as a function of PEM-tip *z*-position. This permits localisation of the PEM-tip to a non-contact *z*-offset position for 2D point scanning (e.g., *z* ≈ 2 μm). Figure 2c–f show the capability to generate high resolution 3D images from single 2D point scans, revealing cellular morphology and 3D-resolved Brillouin frequency shifts that are consistent with previous microscopy measurements on fibroblasts^21^.

To demonstrate the multicellular scalability of the technology and validate the method for future in-vivo applications, *Caenorhabditis elegans* nematodes were PEM scanned (see Methods). Wild type *C. elegans* N2 are unsegmented pseudocoelomate microscopic organisms with large aspect ratios between length and diameter (~1 order of magnitude) and high intrinsic optical transparency (~2% intra-organism variation^23^). Their transparency and well characterised refractive index profile make them an excellent candidate to examine the capabilities of PEM to investigate sub-surface elasticity with high 3D resolution. Initially we employed a strategy of dehydration to suppress nematode movement. However, it is well known that evaporative water loss through desiccation significantly alters the morphology and mechanical properties of nematodes^24^. Figure 2g presents optical, phonon profilometry, and 3D PEM stiffness imaging of the mid-body (pharyngeal-intestinal junction) region of a dehydrated *C. elegans* L2 nematode (ex-vivo, rehydrated for PEM). In comparison, Fig. 2h shows the equivalent measurements from a similar mid-body region of a hydrated anaesthetically immobilised larva. The two states demonstrate a clear deviation in mechanical properties and internal structure, with the hydrated nematode exhibiting maximum 500 MHz shifts in Brillouin frequency (relative to the phosphate buffered saline, PBS, control medium) compared with the 800 MHz shift of the dehydrated specimen (implying greater stiffness) (see Supplementary Note 2, Supplementary Fig. 2, and Methods). Consequently, this reiterates the mechanical and structural modifications brought on by desiccation and motivates the use of anaesthetic immobilisation to preserve native state structure and material properties for the remainder of this study.

**Fig. 3:**
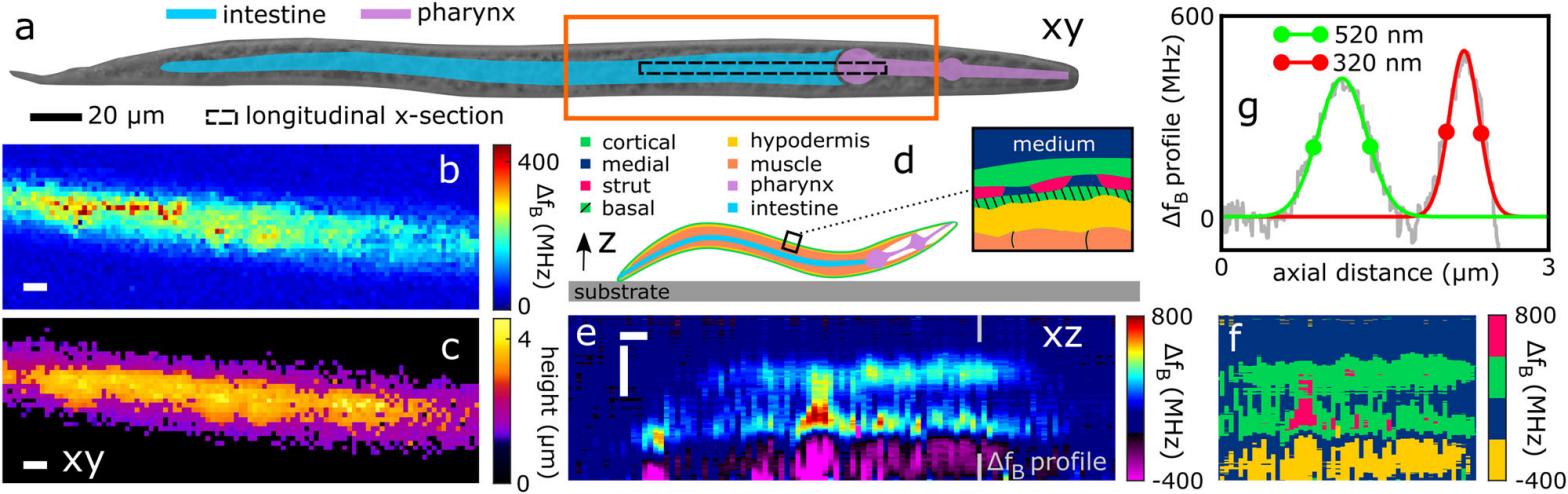
Super resolution depth imaging of the *C. elegans* cuticle in-situ. **a** epi-DF of an L2 larva; orange box indicates the region of interest shown in the PEM 2D stiffness and profilometry maps in **b** and **c**, respectively (101 × 35 pixels acquired at a rate of 2.5 s/pixel in *x*-*y*). **d** Anatomical cartoon of physiological structures (cuticle shown in the inset). **e** Longitudinal stiffness cross section of the nematode indicated by the position of the black dashed box in **a** (101 × 844 pixels acquired in 4 minutes, 3 ms/pixel in *x*-*z*). **f** Segmentation of the Brillouin shifts in (**e**) reveal structures of distinct stiffness that correlate with the cortical/basal, medial, strut, and hypodermis. Horizontal and vertical scale bars are 5 μm and 1 μm, respectively. **g** Experimental measurements of the depth resolved Brillouin shifts along the grey line in **e**. Gaussian fits were used to extract the local thicknesses of the cortical and basal layers (see legend), producing an experimental axial resolution of 320 nm.

The cuticle of *C. elegans* plays a multi-faceted role in protecting the organism from its environment. It is primarily a collagen-based structure which provides structural stability, whilst simultaneously enabling sufficient flexibility for locomotion^25^. The cuticle is not only an extremely important structural component of the *C. elegans* anatomy, but it serves as a specialised model for studying key biological processes shared with vertebrates (e.g., collagen biogenesis, extra-cellular matrix functionality, and organogenesis)^26^. However, new techniques are needed to better understand fundamental physical and mechanical properties of these structures. Cuticle stiffness has been investigated using various contact mechanical deformation based techniques such as atomic force microscopy and microfluidics^25,27^. However, results are often contradictory and these techniques: require significant cuticle processing and isolation, are not 3D resolved, and access stiffness at scales that are either highly surface localised or generalised to the whole organism. Additionally, basic visualisation of sub-surface cuticle structure is exclusively achieved through destructive sectioning and electron microscopy which is known to introduce measurement artefacts. We applied PEM to overcome these challenges in studying cuticle characteristics in 3D without mechanical contact. A hydrated L2 nematode was PEM scanned across the mid-body at the pharyngeal-intestinal junction (Fig. 3a). An initial fast scan demonstrated the acquisition speed of PEM (nominally 50 ms/voxel). A second 250 ms/voxel scan with greater averaging produced the 2D stiffness and profilometry projections in Fig. 3b, c. A detailed discussion on acquisition speed is provided in Supplementary Note 3 and Supplementary Fig. 3, however, in brief, the physical *x*-*y* scanning speed (e.g., for Fig. 3) is on the order of 2.5 s/pixel. However, each *x*-*y* pixel constitutes an entire line-scan in *z* (e.g., 101 pixels long). Therefore, despite the 2.5 s/pixel speed in *x*-*y*, the *x*-*z* scanning speed is on the order of 3 ms/pixel.

To probe the sub-micron thick *C. elegans* cuticle (Fig. 3d) we reduced the TFA *z*-sectioning windows from *z*_*win*_ = 1.28 μm (used in Figs. 1 and 2; see Supplementary Note 4 and Supplementary Fig. 4) to *z*_*win*_ = 520 nm (nominal axial resolutions of 640 nm and 260 nm, respectively) which establishes a compromise between stiffness-precision and axial resolution. The longitudinal stiffness cross-section of the nematode (dashed region in Fig. 3a) is presented in Fig. 3e and reveals several distinct layers that correlate with known cuticle structures: cortical, liquid filled medial, and basal layers, followed by lipid rich hypodermis and sub-hypodermal layers. To ascertain the relative stiffness of the cuticle layers Fig. 3e was segmented into four linearly spaced frequency bins resulting in the cross-section presented in Fig. 3f. Here it is shown that the cuticle layers contain distinct biomechanical properties: PBS-medial Δ*f*_*B*_ = 60 ± 80 MHz, cortical-basal Δ*f*_*B*_ = 300 ± 70 MHz, and hypodermis Δ*f*_*B*_ = -200 ± 70 MHz. Interestingly, the negative Brillouin shift in the sub-basal region (yellow) suggests a −4% decrease in stiffness (see Methods) relative to the control medium which could indicate the presence of fat-storing lipid droplets^22^ which are known to accumulate in both the intestine and hypodermis^28^. Lastly, in Fig. 3e, f there exists a bridge-like structure with a Δ*f*_*B*_ = 600 ± 80 MHz mean Brillouin frequency shift implying that it is 12% stiffer than medium. The geometry and positioning of this feature are consistent with that of a strut, a collagen columnar structure that connects the cortical-basal cuticle layers^29^. The strut’s elevated stiffness relative to its surroundings indicates its mechanical functionality as a load bearing element whilst also providing rigidity to the otherwise flexible cuticle. This important measurement advance could provide the basis of future developmental biology studies capturing time-dependent and disease-related changes in cuticle structure in a label-free and contact-free manner.

**Fig. 4:**
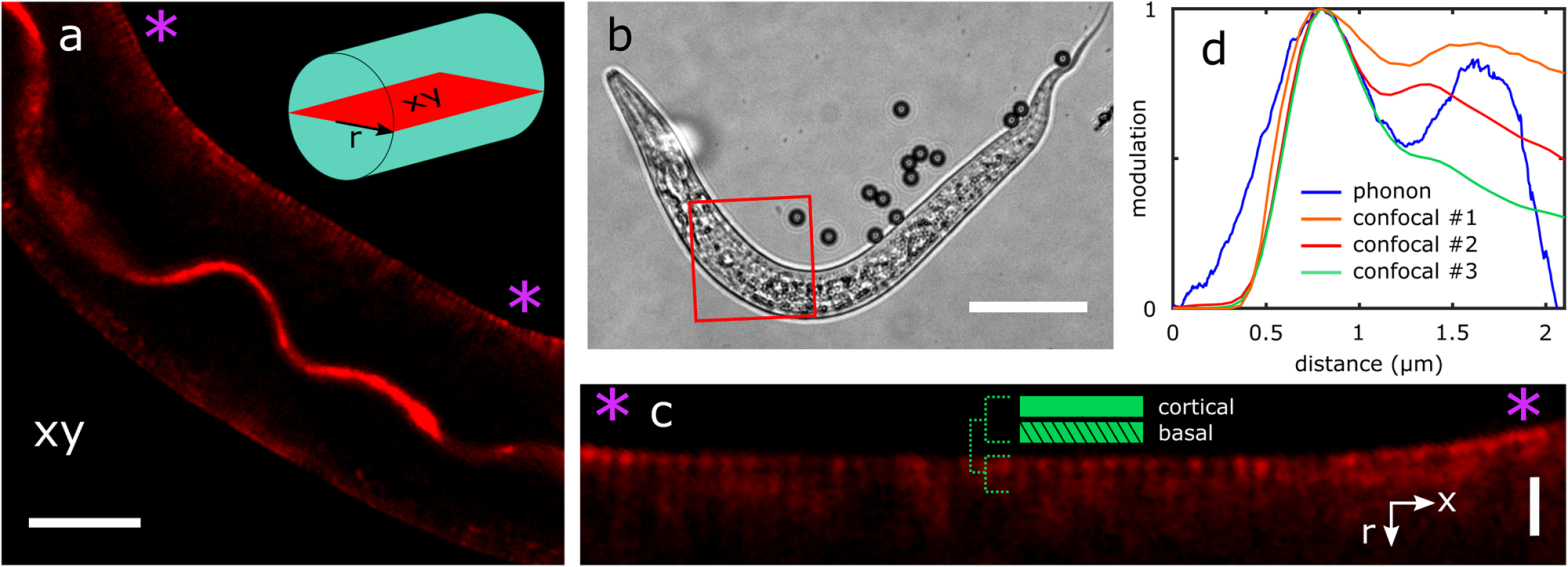
Confocal fluorescence microscopy of *C. elegans* nematode cuticles. **a** Fluorescence intensity map of an L2 nematode (from the region of interest designated by **b**) along the length of the specimen at mid-height (see inset of **a**). The central gastrointestinal tract is illuminated and at the edge of the nematode a stratum of cuticle is observed (**c**) in between the pink asterisks. **d** Average fluorescence intensity modulation cross-sections from 3 nematode specimens. These cross-sections were extracted along cuticle surface normals described in Supplementary Note 5 and Supplementary Fig. 5; 179, 139, and 239 cross-sections were averaged for the orange, red, and green curves, respectively. Note the reduced contrast from confocal microscopy when compared to an equivalent average cross-sectional Δ*f*_*B*_(*z*) profile obtained from PEM imaging the specimen in Fig. 3 (blue line, the average of 225 cross-sections). Scale bars: a) 10 μm, b) 50 μm, c) 2 μm.

To the best of our knowledge, PEM is the first method that can resolve the thicknesses of these layers in-situ (Fig. 3g). The axial resolution (Supplementary Note 4) of the technique is realised through measuring the basal thickness (at the position of the grey line in Fig. 3e) down to 320 nm. Next we employed confocal laser scanning microscopy and a lipophilic fluorophore to corroborate the morphology of the cuticle layers observed with PEM imaging. The mid-body regions of 3 anesthetised nematode specimens were scanned with high 3D resolution (~15–60 min each) according to the methodology in Methods (Fig. 4a–c). Fluorescence intensity cross-sections perpendicular to the direction of the cuticle were extracted from the resulting data and this process is described further in Supplementary Note 5 and Supplementary Fig. 5. The cuticle fluorescence cross-sections from each of the 3 specimens were averaged into 3 cuticle-morphology profiles (see Fig. 4d). Figure 4d demonstrates that there is strong qualitative agreement between the two techniques and it is apparent that our PEM imaging modality offers enhanced contrast, while maintaining very high resolution without fluorescent labels and high numerical aperture lenses.

Direct quantitative agreement between these two different techniques is not expected due to a number of factors: (a) mismatched gradients between mechanical properties and dye-conjugation; (b) variation in nematode size and age; (c) inter- and intra-specimen variations in fluorophore uptake and emission efficiency (low contrast and specificity); (d) cuticle thickness heterogeneity; and (e) differences in the nature of the background signals for each technique. These challenges lead to variability in the low contrast confocal cross-sections measured in Fig. 4d. Regarding (e), background signals in our PEM technique come in the form of Brillouin shift measurements at the frequency of the couplant medium, which have been subtracted from all measurements presented here. This facilitates relatively trivial measurements of the peak widths, such as in Fig. 3g where the green and red distributions are fully resolved since the region between them falls to the background level (Δ*f*_*B*_ = 0). However, in confocal microscopy the background fluorescence from regions surrounding the cuticle (yet at the same *z*-plane) produce strong contributions to the fluorescence cross-section profiles, ultimately shrouding cuticle-specific measurements. Despite these challenges, the two techniques reveal similar cuticle morphologies and spatial scales which demonstrates the promise of PEM for resolving sub-micron structures in biology with very high contrast.

## Discussion

The fully optical fibre-based PEM system presented in this work was capable of measuring Brillouin frequency shifts with precision on the order of 7 MHz (*n* = 101 measurements in water) and 160 MHz frequency resolutions, which are comparable to current state-of-the-art spontaneous (10 MHz and 250 MHz, respectively)^30^ and stimulated (7 MHz and 151 MHz, respectively)^31^ Brillouin microscopy systems. The above spectral precision of the PEM considers the frequency measurement of the entire time-domain signal. However, for the PEM it is appropriate to characterise the spectral precision as a function of axial distance, since the time signal represents the axial spatial dimension - i.e., instantaneous changes in signal frequency correspond to depth variations in Brillouin frequency. In addition to signal averaging, the depth precision will also be a function of the window-width used in time-frequency analysis (discussed further in Supplementary Note 6). We report that within 4 μm of depth, the PEM is capable of measuring instantaneous frequency with precision in the range of 28–52 MHz and 11–18 MHz for the wavelet window-widths used (see Supplementary Fig. 6). The decrease in precision as a function of axial distance is due to the depth-dependence of the signal-to-noise ratio as the propagating phonon attenuates^9^, yet does not affect the axial resolution of the technique. To ascertain the lateral resolution of the PEM, the edge of an arbitrarily large bar on a US Air Force resolution target was scanned revealing an effective point spread function of approximately 2.05 ± 0.19 μm (see Supplementary Fig. 7 in Supplementary Note 7). High numerical aperture lenses used for Brillouin microscopy permit lateral resolutions on the order of ~0.5 μm, nevertheless our Brillouin fibre-probe represents a key step for the field without severely compromising lateral resolution and yet providing an order of magnitude greater axial resolution than any Brillouin microscopy technique (indeed including optical endo-microscopy).

PEM technology offers super-optical resolution in the axial dimension utilising sub-optical wavelength coherent acoustic phonons. In theory, these same sub-optical wavelength CAPs can be used as the lateral resolution mechanism, provided that the transducer on the fibre-tip is converted into an acoustic lens^32,33^, which would make possible full 3D sub-optical resolution imaging. Realising this technology would present a benign path to super-optical resolution imaging since it uses non-destructive phonons and low-energy near infrared (NIR) photons. GHz frequency acoustic phonons carry five orders of magnitude less energy than photons with the equivalent wavelength (e.g., near ultraviolet light). Additionally, the NIR probe light used in this work (~10^4^ W/cm^2^) was ~8 orders of magnitude lower photon dosages than previously reported experimental cellular-damage thresholds (~10^12^ W/cm^2^)^34^. Currently, the PEM is capable of ~6 μm depth measurements, which can be extended to 20–40 μm by using IR-MIR probe wavelengths (depth measurement range $\propto {\lambda }_{probe}^{2}$). However, the depth range is exchanged for an unparalleled 320 nm axial resolution which is achieved without confocally scanning the device, and without the risk of depth exposure-dependent fluorophore bleaching. This characteristic of PEM permits full 3D imaging with acquisition speeds of 50 ms/voxel (comparable to current in-vivo biomechanical 3D microscopy^12^), despite its current 0.5 s/pixel lateral scanning speed (Supplementary Note 3). The latter restricts current measurements to fixed cells and immobilised nematodes, however, with PEM a single line scan (e.g., in *x*) constitutes a full 2D cross-sectional map (*x*-*z*) with 3 orders of magnitude faster per-pixel acquisition speed than *x*-*y* (Fig. 3b, e). Improved transducer efficiency and parallelised fibre bundle^20^ scanning techniques will increase lateral acquisition speed by 1–2 orders of magnitude rapidly scaling PEM towards real-time measurements.

Fibre bundle implementation of the PEM also offers a path towards full endoscopic implementation, as it permits static scanning, i.e., maintaining a static position at the distal end while the pump and probe beams are sequentially scanned through the bundle cores at the proximal end^20,35^. Alternate scanning configurations based on resonant vibration of single optical fibres have been used in optical and coherent Raman endoscopic imaging applications that could also be applied for endoscopically implementing PEM^36,37^. However, it is worth noting that future applications for our fibre technology are not limited to spatially resolved imaging as demonstrated with our proof-of-concept hypodermic needle-delivered Brillouin fibre spectrometer^38^.

In summary, this report has demonstrated the first endoscopically viable imaging of biomechanical properties – through the phenomenon of Brillouin light scattering – both on single-cell and complex whole organism scales. More generally, we have shown the potential for high resolution and high contrast 3D endo-microscopy on optically passive biology that does not rely on fluorescent, photoacoustic, or non-linear optical properties. We anticipate that the non-contact and sub-surface 3D imaging capabilities of this novel technology could enable new breakthroughs in cell and tissue biology in an in-vitro environment. Beyond this, PEM makes possible the application of mechanobiology – currently led by non-endoscopically viable technologies such as atomic force microscopy – to in-human measurements towards clinical applications in optical biopsies and in-vivo histopathology for early disease diagnosis^38^.

## Methods

### Time-resolved Brillouin scattering

TRBS is a picosecond laser ultrasound technique whereby a pump laser pulse is absorbed by an opto-acoustic transducer; thermal expansion creates a broadband coherent acoustic phonon (CAP) field which propagates through the sample. The CAP acts as a weak mirror due to the photo-elastic effect and is detected via a time-delayed probe laser pulse. Reflected probe light from both the transducer interface and the acoustic wavefront are collected by the same optical fibre used to deliver the beams and detected by a photodiode (ref. and scat. in Fig. 1b).

To recover the Brillouin frequency from the time-dependent reflectivity, consider that the path length difference between the ref. and scat. components leads to a phase difference that grows as the acoustic pulse propagates away from the transducer,

$$\Delta \phi =2k\Delta z=2kvt$$

where *k* is the optical wavenumber within the control medium 2*πn*/*λ*_*probe*_, and Δ*z* is the path length difference, itself determined by the product of the time-of-flight (ToF) signal time-base *t* and velocity *v* (see the ‘Signal processing’ section). Assuming linear propagation of the acoustic pulse in a homogeneous medium, the signal collected will thus have an oscillation at frequency^7^,

1 $${f}_{B}=\frac{1}{2\pi }\frac{d\Delta \phi }{dt}=\frac{2kv}{2\pi }=\frac{2nv}{{\lambda }_{probe}}$$

where we recover the familiar expression for the Brillouin frequency shift (assuming normal probe incidence). For samples with homogeneous refractive index, a measurement of the Brillouin frequency shift infers a measurement of the local sound velocity. Taking the quantification of mechanical properties further, if the mass density, *ρ*, is known, measured, or approximated, the longitudinal modulus of the specimen can be determined. The complex longitudinal modulus (M^*^) describes both the storage and dissipation of energy during a uni-axial compression, and can be written as^5,39,40^

2 $${M}^{* }({f}_{B})={M}^{{\prime} }+i{M}^{{\prime\prime} }={v}^{2}\rho +i\frac{\alpha \rho {v}^{3}}{\pi {f}_{B}}$$

Thus in addition to measuring the frequency of the signal, measuring the depth attenuation coefficient, *α*, of the phonon wavefront - either through fitting a decaying exponential to the time-domain signal^20^ or measuring the frequency bandwidth of the TRBS ToF signal - provides access to the longitudinal loss modulus *M*^*″*^ which describes energy dissipated during the compression.

It is worth noting that in this work we have used the relative Brillouin frequency shift (compared with the control medium) as a proxy for stiffness. We are reporting frequency shifts of *C. elegans* anatomical structures and materials (such as intra-cuticle layers) that occupy 1 μm^3^ volumes, whereas current refractive index and mass density data^23^ (which would enable conversion to longitudinal modulus) have been obtained by averaging over an entire 10^5^ μm^3^ organism volume. To the best of our knowledge, the refractive index and mass density of subcellular scale cuticle constituents have not been previously characterised, and so we approximate stiffness through the measured sound velocity (Eq. (1)) using constant values^23 ^*n* ≈ 1.38 and *ρ* ≈ 1200 kg/m^3^.

### Optical fibre-based opto-acoustic transducer

The imaging fibre probe utilises a custom-made single-mode fibre patch cable (Thorlabs, 780HP) with an FC/PC connector at one end and bare-fibre at the other. It has a 5 μm diameter core, 125 μm diameter cladding, a numerical aperture of 0.13, and a centre wavelength of 780 nm. The bare end of the fibre is stripped, cleaved, and coated with an opto-acoustic transducer. To create the transducer, a DC magnetron sputter-coater (HHV BT3000) is used to deposit two layers. The first layer, which is 5-nm-thick and made of Indium Tin Oxide (ITO), provides adhesion. The second layer, which is 15-nm-thick and made of gold (40 mA plasma current), functions as an opto-acoustic transducer and generates the reference beam needed for TRBS (ref. in Fig. 1b). The thickness of the gold layer is carefully selected as a compromise between pump absorption (15%), probe transmission (40%) for Brillouin scattering detection, and transducer damage threshold^41^. The thickness of the layers is determined using white-light transmission spectroscopy on a calibration sample (glass coverslip) coated simultaneously with the fibre. The calibration sample’s transmittance is fitted to a one-dimensional model of the layer stack, created using the matrix transfer method and the refractive index reported by Ciesielsky et al.^42^.

### Optical and electronic systems

The phononic endo-microscopy system (Fig. 1a) is built around a dual Ti:Sapphire (Tsunami Spectra-Physics) laser asynchronous optical sampling system (ASOPS) with 100 *fs* pulses at 80 MHz repetition rate. This allows the timing of the laser pulses to be precisely controlled and for the time delay between the pulses to be swept from 0 to 12.5 ns every 100 μs (10 kHz delay rate) with sampling periods on the order of ~3 ps.

Pump and probe wavelengths of 780 nm and 830 nm, respectively, were primarily used for this work (both lasers are tunable within the range 700–900 nm). Coupling from free-space to fibre was accomplished with fibre ports (Thorlabs, PAF-X-15-PC-B), which then relayed through a custom wavelength division multiplexer (OZ Optics, 800 nm wavelength dichroic edge) to maximise coupling efficiency into a single common channel. A fibre optic circulator (OZ Optics, 780 nm centre wavelength) was used to maximise the signal-to-noise ratio of the Brillouin scattered reflected light that counter-propagates through the system (see Fig. 1a). The final common channel consists of a single-mode custom patch-cable (described in the ‘Optical fibre-based opto-acoustic transducer’ section) which is exposed to a total average power of ~15 mW.

The TRBS ToF signal - i.e. the interference of light reflected from the glass/gold interface (ref. in Fig. 1b) and reflected Brillouin scattered light from the acoustic wavefront (scat. in Fig. 1b) - is then detected on a Si-switchable gain detector (Thorlabs, PDA36A-EC), amplified (Mini-Circuits, ZFL-500+), low-pass filtered (Mini-Circuits, BLP-7-75+) and acquired on a digital oscilloscope (Lecroy, WaveRunner HRO 66Zi). The effective bandwidth of the detection system is generally dictated by the detector bandwidth (5.5 MHz at 10 *dB* gain) scaled by the ASOPS scaling factor (80 MHz⋅100 μs = 8000): 44 GHz.

### 3D phononic imaging protocol

To initiate phononic endo-microscopy, a specimen is first located using the ancillary optical imaging system (Supplementary Note 8 and Supplementary Fig. 8). Next, the fibre probe (held in place above the sample with a fibre clamp) is descended to the approximate position of the optical system’s focal plane using a *z*-micrometre translation stage (mounted vertically). Once the fibre-tip has entered the extended depth of focus of the imaging system, a piezoelectric nanopositioner (Physik Instrumente, P-721.OLQ) (connected in-series with the fibre clamp) is activated to enable fine control of the fibre-tip *z*-position (100 μm travel range, with 0.5 nm step resolution). In a manner similar to deflection feedback in atomic force microscopy (AFM), the nanopositioner is progressively activated (increasing applied voltage) until a shift in the baseline depth-resolved Brillouin frequency shift is observed, e.g. using a Fourier or wavelet transform approach (as in Fig. 2a, b. For example in Fig. 2b, when the HeLa cell enters the depth measurement range of the PEM, the depth-resolved frequency measurement experiences a rise in frequency toward the end of the signal window (transitioning from the cyan line to the light green lines in Fig. 2b). The user then selects a stand-off distance for the fibre-tip, e.g. based on the expected height of the specimen considering the depth measurement range of this current PEM set-up (6 μm with *λ*_*probe*_ = 830 nm). This is analogous to considering the height of a specimen relative to the dimensions of the cantilever and tip in AFM. However, unlike AFM, the PEM can make measurements (without artefacts) on objects that are taller than the depth measurement range (e.g. set by the cantilever and tip dimensions in AFM) since contact/tapping are not required and the piezo nanopositioner and *z*-micrometre have translation ranges of 100 μm–25 mm. Once an object has been localised optically and axially, the specimen is scanned laterally relative to the stationary PEM-tip using high speed motorised scanning stages (Thorlabs, MLS203-1). At each lateral scan position, a ToF signal is collected, in which the time-domain of the signal corresponds to the axial spatial domain, thus eliminating the need for confocally scanning the specimen or fibre to accomplish 3D imaging.

### Signal processing

The ToF signals were processed offline using custom written MATLAB (Mathworks, R2022b) software and were subjected to the following signal processing. The signals consist of 1) a coincidence peak (at signal time *t* = 0) caused by electron dynamics, 2) a slow-decaying thermal response, and 3) the TRBS ToF. The TRBS ToF oscillations (Fig. 1c) are extracted from the thermal background by polynomial fit subtraction and the peak frequency in the Fourier domain is found using the fast Fourier transform (FFT) algorithm (Fig. 1d) which represents a measurement of the Brillouin frequency shift. Using Eq. (1), and a priori information regarding the refractive index (e.g. *n* = 1.33 for the phosphate buffered saline medium), a measurement of the Brillouin frequency can be translated into a measurement of the local sound velocity of the specimen. This then allows us to cast the time domain of our ToF signals into the axial spatial domain via the relationship *z* = *vt* which permits both 3D stiffness imaging and profilometry.

### Depth-resolved signal processing

With the PEM system, mechanical heterogeneity (on scales larger than the acoustic wavelength) in the axial direction manifests as changes in the instantaneous frequency of the ToF signal (Fig. 1c) which can be extracted through time-frequency analysis (TFA), such as the continuous wavelet transform (CWT):

3 $$C(f,t)=\int\nolimits_{-\infty }^{\infty }ToF(t)\cdot \Psi (f,t)dt$$

where the signal of interest is convolved with a *mother wavelet*, Ψ, which is a function that occupies a finite window in time. The frequency (*f*) and time-position (*t*) of this function is varied, leading to a set of daughter wavelets. We used the complex morlet as the mother wavelet:

4 $$\Psi (f,t)=\frac{1}{\sqrt{\pi {f}_{b}}}\exp \left(2\pi ift-\frac{{t}^{2}}{{f}_{b}}\right)$$

where *f* is the centre frequency (e.g. varied around the expected *f*_*B*_ Brillouin frequency shift), and *f*_*b*_ is a bandwidth parameter related to the width of the wavelet in wavelengths or periods. The output of the transformation is a set of coefficients *C*, representing the signal amplitude as a function of frequency and signal time/axial distance (Eq. (3)), and can be visualised in the form of a spectrograph (Fig. 1e) or the amplitude response at a single frequency^20^.

Topographic information about the specimen can also be obtained from the output of the CWT. We accomplish this by measuring the proximity between the specimen and the fibre-tip, i.e. centroiding the amplitude roll-off at the Brillouin frequency shift of the control medium (e.g. PBS)^20^. This process is physically described by the instantaneous change in sound velocity (with depth) between the control medium and the depth-resolved specimen, which is accompanied by the probe laser beam Brillouin scattering with different phonon frequencies in the two media. Following from Eq. (3), this transition is measured through the following relationships:

5 $${C}^{{\prime} }(f,t)=\hat{A}\cdot \hat{C}$$

6 $${C}^{{\prime} }[{z}_{p}({v}_{med},t)]=\frac{1}{2}(1+{\hat{C}}_{min}^{{\prime} })$$

where $A=\exp [{\alpha }_{med}{v}_{med}t]$ represents the signal decay due to acoustic attenuation in the control medium, and $\hat{A}$ and $\hat{C}$ represent normalisation by the respective maximum values, e.g. $\hat{A}=A/{A}_{max}$. The acoustic attenuation of the control medium is determined by acquiring a series of ToF signals when the PEM-tip is positioned far away from a specimen but is still immersed in the control.

When using CWT to measure the Brillouin frequency shift in three dimensions, the axial resolution of PEM will be determined by the spatial extent of the wavelet. For instance, given an acoustic wavelength of *λ*_*acoustic*_ = *λ*_*optical*_/2*n* = 312 nm, a wavelet defined by a width of approximately 1.6 acoustic wavelengths (*N*_*λ*_ ≈ 1.6) gives rise to a wavelet full-width half-maximum of 520 nm (Supplementary Note 4), and a theoretical axial resolution^21^ of *r*_*ax*_ ≈ *N*_*λ*_*λ*_*acoustic*_/2 ≈ 260 nm.

A final consideration is made for temperature fluctuations that surround the PEM-tip. Thermo-optic and thermo-acoustic affects cause minor background fluctuations in the instantaneous depth-resolved Brillouin frequency shift measurements (Eq. (1)). These are stable over the time scales of single experiments and therefore can be compensated in the following way. A series of reference signals are accumulated without the influence of a specimen (e.g. with the PEM-tip immersed in the PBS medium). The depth-resolved Brillouin shift from ~100 such measurements are averaged together. These provide a reference frequency measurement (${f}_{B}^{0}$) with which a relative Brillouin shift is calculated, i.e. $\Delta {f}_{B}(z)={f}_{B}(z)-{f}_{B}^{0}(z)$ while also compensating for the effect of temperature fluctuations in each PEM 3D image presented in this work. As a result, the cross-sections presented in Figs. 2e–h and 3e do not utilise binary masks to suppress Brillouin frequency shifts from the environment surrounding the specimen. An alternative method for background subtraction via segmentation is used in Supplementary Note 2 whereby axial slices are segmented and the background frequency of each slice is used to correct for any depth-dependent variation in background Brillouin frequency.

### Specimen preparation

#### Cells

Human bone fibroblast cell line MG-63 (ATCC; CRL-1427), human cervical epithelial cell line HeLa (ATCC; CCL-2), and mouse embryonic fibroblast cell line NIH/3T3 (ATCC; CRL-1658) were used for this work. These were all maintained in DMEM (Merck, D6421) supplemented with 2 mM L-glutamine (Merck, G7513), 10% FBS (Merck, F9665), 50 μg/mL penicillin and streptomycin (Merck, P0781). Cells of passage numbers 20–35 were incubated in a humidified atmosphere containing 5% CO_2_ at 37 °*C*. Polystyrene petri dishes (35 mm) were coated with PLL (Merck, P04707) as described by the manufacturer to aid cell adhesion. Cells were seeded at a density of 10^4^ cells per dish. After fixation for 10 min with 4% formaldehyde solution (Merck, 1.00496) cells were stored and imaged in phosphate buffered saline solution (Merck, D8537). All cell lines tested negative for mycoplasma contamination as tested using MycoAlert mycoplasma detection kit (Lonza; LT07-318).

#### C. elegans nematodes

Nematodes were purchased from Caenorhabditis Genetics Center University of Minnesota. All other reagents were purchased from Sigma-Aldrich unless otherwise stated. Nematode growth and maintenance: *C. elegans* nematodes (Bristol N2) were maintained on nematode growth medium (NGM) agar and *E. coli* (OP50) at 20 °C. NGM Agar was produced using sodium chloride (1.5 g), agar (8.5 g) and peptone (1.25 g) made up with deionised water (1 L) and autoclaved. Calcium chloride (0.5 mL, 1 M), cholesterol in ethanol (0.5 mL, 5 mg/mL), magnesium sulphate (0.5 mL, 1 M), and potassium buffer solution (12.5 mL 1 M) were added to molten agar poured into petri plates and were allowed to set and dry overnight. *E. coli* OP50 pure stock cultures (in 10% glycerol, stored at –80 °C) from the –80 °C freezer were thawed then added to lysogeny broth (LB, 50 mL) media and allowed to grow (8 h, 37 °C, 180 rpm). Freshly grown *E. coli* cultures (300–1000 μL) were pipetted in NGM petri dishes containing NGM Agar and spread across its surface. Plates were incubated (37 °C) until a bacterial lawn was visible and then stored until use (4 °C). Synchronised growth cycles of *C. elegans* were prepared by harvesting eggs from gravid females. Gravid nematodes were collected by rinsing a NGM growth plate with sterile deionized water (3.5 mL). Sodium hydroxide (5 M, 0.5 mL) and sodium hypochlorite (5%, 1 mL) were added to the worm suspension and vortexed (10 min) to separate nematodes from the eggs. The eggs were collected using centrifugation (1500 rpm, 1 min), and subsequently washed with 5 mL of sterile deionized water (1500 rpm, 1 min). The centrifuged egg suspension was aspirated to 0.1 mL and plated onto a fresh plate of NGM agar, seeded overnight with an *E. coli* lawn. The generation time of *C. elegans* using these conditions was 3 to 4 days. Life-cycle isolation: *C. elegans* lifecycle isolation was achieved using filtration of synchronised nematodes. At day 5 mixed populations of adult *C. elegans* and L1-L2 larvae, were collected from NMG synchronised plates in sterile deionized water (5 mL, 18.2 Ω). Nematode suspensions were separated using a Merck 20 μm Nylon net filter. L1-L2 larvae was isolated from the filtrate whereas and adult nematodes were deposited on the top of the filter. Adult *C. elegans* and L1-L2 larvae were separately washed in deionised water and centrifugation (15 mL, 1500 rpm, 3 times) and maintained at 4 °C, until further use. Preparation of *C. elegans* for PEM analysis: L1-L2 larvae were deposited drop wise in distinct populations on a petri dish (35 mm). Excess deionised water was aspirated using a pipette and blotted away with filter paper. The petri dish was filled with PBS as a hydrating medium and the nematodes were anesthetised with sodium azide (10 mM). This was essential for the worms to be maintained in a hydrated and immobilised state for the duration of PEM analysis.

### Confocal microscopy

Fluorescence imaging was performed with a Zeiss LSM900 microscope with Airyscan 2 detector, using 20×, 0.80 NA and 100×, 1.30 NA objective lenses. *C. elegans* L1-L2 larvae were isolated as described in Methods before staining with Di-l lipophilic dye for three hours following the protocol described by Schultz and Gumienny^43^. After staining, worms were deposited onto an agar disc and a coverslip was placed on top. Once on the microscope, nematodes were found using a combination of widefield fluorescence and brightfield microscopy at 20× magnification. Once regions of interest had been identified, the objective lens was changed to the 100 × oil immersion lens to achieve a lateral resolution of ~300 nm (estimated using the Rayleigh criterion 0.61*λ*/*NA* for emission wavelength *λ* = 565 nm). Z-stacks were acquired for regions near the mid-body of each nematode, and the plane through the centre of the worm was chosen for further analysis.

#### Statistics and reproducibility

A sample size of *n* = 5 mammalian cells (1 HeLa, 2 3T3 mouse fibroblasts, and 2 MG-63 cells) and *n* = 3 *C. elegans* larvae were used without sample size calculation. This was to demonstrate proof of concept 3D imaging for our new technology. *n* = 3 *C. elegans* larvae were imaged using confocal fluorescence microscopy to reveal the existence of cuticle structure. The data associated with manuscript Fig. 3 was scanned twice to demonstrate repeatability for different scanning speeds (see Supplementary Fig. 3). Phononic imaging organisms were selected randomly; confocal imaging organisms were selected to have similar nematode diameter compared with phononic imaging organisms.

### Reporting summary

Further information on research design is available in the Nature Portfolio Reporting Summary linked to this article.

### Supplementary information

Supplementary information

Description of Supplementary Materials

Supplementary Data 1

Reporting Summary

## Data Availability

The source data behind the graphs can be found in Supplementary Data 1. All other data are present in the manuscript or can be obtained from the authors on reasonable request.
