# Supplementary material for: Label-free Brillouin endo-microscopy for the quantitative 3D imaging of sub-micrometre biology
Source: Commun Biol. 2024 Apr 15;7:451. doi: 10.1038/s42003-024-06126-4 (PMC11018753; doi:10.1038/s42003-024-06126-4)
Supplement: Supplementary file 1 — Supplementary information [file 42003_2024_6126_MOESM1_ESM.pdf]

Supplementary information for: Label-free Brillouin endo-microscopy for the quantitative 3D imaging of sub-micrometre biology

Supplementary Note 1

The acoustic spectrum produced through the photoacoustic effect is known to be a function of both the optical absorption depth in and the thickness of the photoacoustic generation material (in addition to the material's sound velocity). The former dictates the thermal stress profile, and therefore the temporal/spatial extent of the resulting strain pulse (i.e. its frequency content).[44] The latter determines the resonant modes of the acoustic cavity created by the transducer.[45] In this work, the photoacoustic generation material is a gold transducer film. For near-bulk thicknesses (e.g. on the order of 200 nm), a bipolar strain pulse is generated within the gold film (upon pump pulsed laser irradiation) due to the acoustic reflection at the gold/glass-substrate interface. Consistent with Ref.[44] this produces a broadband phonon field with a peak amplitude at a frequency

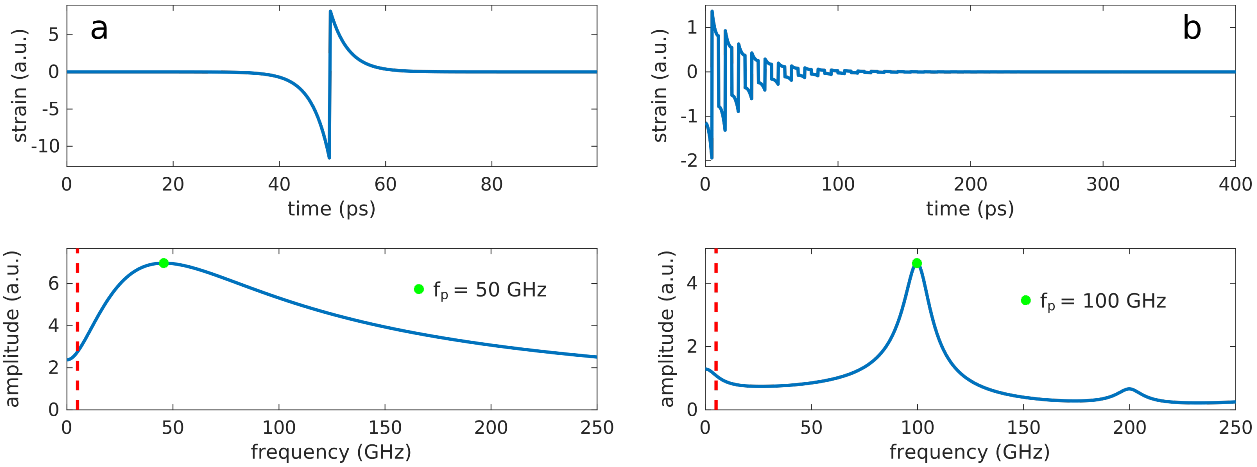

Supplementary Figure 1 a-top) Simulated bipolar strain pulse profile propagating through water (~40 ps after photoacoustic generation in a 200 nm thickness gold transducer), and the corresponding frequency spectrum (bottom) which contains a 50 GHz frequency at peak amplitude and a 100 GHz 3dB bandwidth. b-top) When using a thin-film (e.g. 20 nm of gold), energy in the acoustic spectrum is shifted to the cavity modes of the transducer (e.g. the fundamental and first harmonic in the frequency spectrum in b-bottom) which contains a peak amplitude at 100 GHz and a 20 GHz 3dB bandwidth. The red dashed line represents the region of the acoustic spectrum in which our time-resolved Brillouin scattering interaction takes place (5 GHz).

of approximately 50 GHz and a 3dB bandwidth of approximately 100 GHz. This response is confirmed through a picosecond ultrasonic simulation based on the work of Ref.[46] and is shown in Supplementary Figure 1a. In this work we have used a 20 nm thickness gold thin-film transducer to facilitate optimal optical transmission of the NIR probe bream. This transmission optimisation comes with the trade-off of mechanical resonances that are out-of-band with the $f_B = 5\ GHz$ signal of interest, with a phonon spectrum peak at approximately 100 GHz and a 3dB bandwidth around this peak of approximately 20 GHz (see Supplementary Figure 1b).

Supplementary Note 2

To further examine the Brillouin frequency shifts of the nematodes, the frequency shift distribution was plotted as shown in Supplementary Figure 2a. Each distribution shows a large peak at the background frequency, which differs slightly between the two datasets due to different probe wavelengths and transducer variability. Additionally, the background frequency varied weakly with depth due to local heating at the transducer. To remove the effect of differing background frequencies, the Brillouin shift relative to background was used. To do this, the volume was segmented into "worm" and "background", which is explained in detail below.

First, each slice was used to generate a binary mask of values above some threshold. One threshold, chosen manually, was used for both hydrated datasets and a different threshold for the dehydrated datasets due to the change in Brillouin shift with probe wavelength (830 nm for hydrated and 780 nm for dehydrated). Next the binary mask was dilated and eroded using MATLAB's *imdilate* and *imerode* functions to remove erroneous (high-frequency) background pixels and to smooth the edge of the binary mask. This ensured minimal contribution of background and inclusion of voxels at the edge of the worm which would have been excluded due to the non-smooth

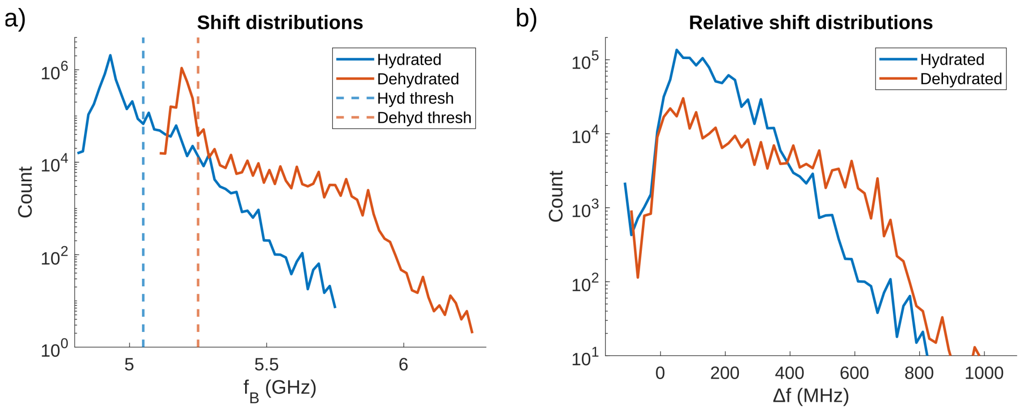

Supplementary Figure 2 a) Brillouin frequency shift and b) Brillouin frequency shift relative to background (Δf_B, Eq. 1) distributions for hydrated and dehydrated nematodes. Segmentation thresholds used to generate initial binary masks are shown as dashed lines on a); the two distributions have different background frequencies due to different probe wavelength and transducers. Shift relative to background reveals that dehydrated nematodes are stiffer and more heterogeneous.

edge of the initial binary mask.

The binary mask was then used to segment the slice into “worm” and “background”, and the mean of the background voxels was subtracted from each worm voxel to obtain the Brillouin shift relative to background,

$$\Delta f_B = f_B^{worm} - f_B^{background}, \tag{1}$$

the distribution of which is plotted in Supplementary Figure 2b.

Using relative frequency shift allows comparison of measurements with different transducers and probe wavelengths as shown in Supplementary Figure 2b. Comparing the distributions, $\Delta f_B = 138 \pm 95\ MHz$ (mean $\pm$ standard deviation) for hydrated nematodes and $\Delta f_B = 199 \pm 180\ MHz$ for dehydrated nematodes. This is interpreted to mean that hydrated nematodes are softer and more homogeneous than dehydrated nematodes.

Supplementary Note 3

The acquisition speed of the phononic endo-microscopy (PEM) system is determined by the amount of signal averaging performed on the oscilloscope (partially dictated by the $\sim 10^{-5}$ time-resolved Brillouin scattering efficiency) and the asynchronous optical sampling mechanism discussed in Methods. Using Fig. 3b as an example, 5,000 averages were acquired for each pixel in the 2D point scan which amounted to $\approx$2.05 $s/pixel$ (in x-y). However, since a single 2D PEM scan produces a 3D-resolved data-cube (the time-domain of each time-of-flight signal represents a line-scan in the axial spatial dimension), the per x-z pixel and per-3D-voxel imaging speeds are much faster than the x-y per-pixel speed. Fig. 3e spans 3.3 μm of axial space; using the 320 nm axial resolution we have demonstrated experimentally, we conservatively approximate that the cross-section contains 10 fully depth-resolved z-pixels. Therefore, the effective imaging speed of the PEM in this case is 250 $ms/voxel$. To demonstrate faster acquisition speed, a second PEM scan was performed over the same region of interest (Supplementary Figure 3a-b) with 1,000 oscillo-

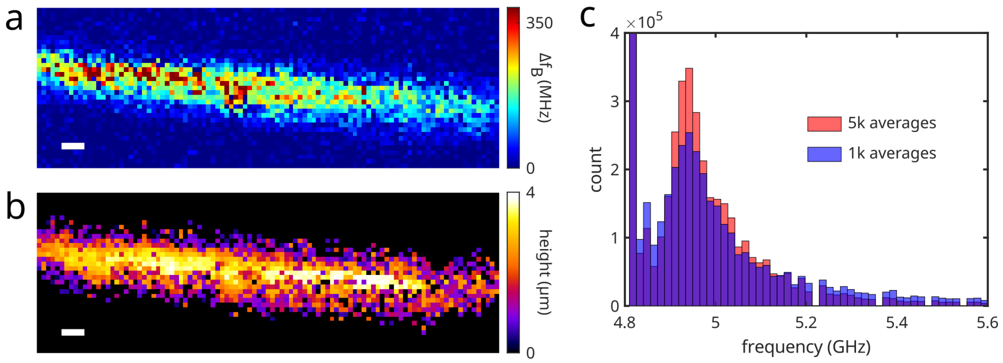

Supplementary Figure 3 a-b) 2D Brillouin frequency shift and phononic profilometry maps as measured by the PEM system with 1,000 oscilloscope averages (50 $ms/voxel$) which reproduce the measurements and variations seen in Fig. 3b-c (acquired with 5,000 averages, 250 $ms/voxel$). c) Histograms of the 3D frequency measurements made at the two acquisition speeds demonstrate the fidelity of the system at 1,000 averages despite containing half the SNR of the 5,000 averages case.

scope averages (≈0.5 $s/pixel$ in x-y), which translates to a 50 $ms/voxel$ 3D-imaging speed, albeit with half the signal to noise ratio ($\sqrt{5^{-1}}$). Despite lower SNR, the viscoelastic information within the 3D data-cubes obtained between the two scans are highly comparable as shown by the histograms in Supplementary Figure 3c. Future advancements in transducer design and non-ASOPS sampling will push PEM toward video-rate acquisition speed without sacrificing SNR.

Supplementary Note 4

Axial resolution (see Eq. 2) for the PEM system is determined by half the acoustic wavelength (like in optics with the optical wavelength) and the width of the time-window used for time frequency analysis (as opposed to the numerical aperture squared as in optics), which in this work was the continuous wavelet transform.

$$r_z = \frac{N_\lambda \lambda_{acoustic}}{2} \tag{2}$$

This effective axial point-spread function is shown in Supplementary Figure 4 for the cases of $\times$1.6 and $\times$4 acoustic wavelengths ($\lambda_{acoustic} = 312$ nm in water) window widths. The widths of the windows were measured at the positions of the full width at half maximum which were 520

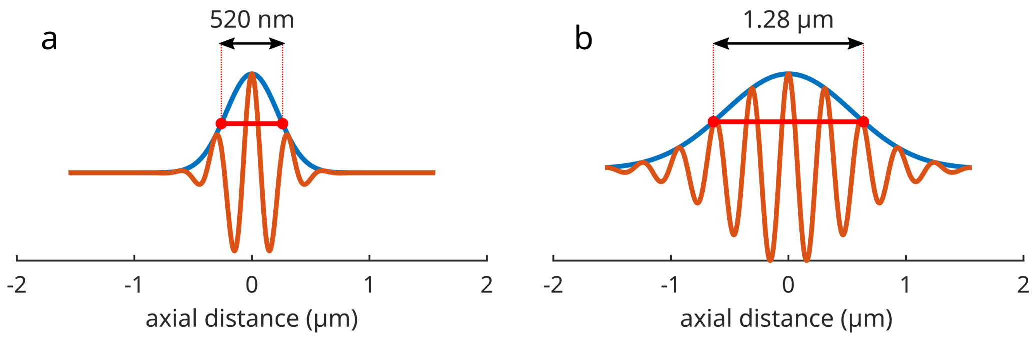

Supplementary Figure 4 a) A daughter wavelet (orange) containing an envelope (blue) that is approximately 1.6 acoustic wavelengths ($\lambda_{acoustic} = \lambda_{probe}/2n \approx 312$ nm in water). This sets the axial resolution obtainable with this wavelet which is approximately half the full width at half maximum value: 260 nm. b) Comparatively, a daughter wavelet with a width of $4\lambda_{acoustic}$ enables nominal axial resolutions of 640 nm.

nm and 1.28 μm respectively. To estimate the axial resolutions of these effective point-spread functions, half the window width is considered,[21] giving nominal values of 260 nm and 640 nm respectively. Increasing the width of the daughter wavelet worsens the axial resolution of the system yet improves the precision on the frequency measurement (Supplementary Figure 6) by way of the uncertainty principle. As such, the $4\lambda_{acoustic}$ case was used to provide more precise viscoelastic measurements in Figs. 1-2, while the $1.6\lambda_{acoustic}$ case was used to demonstrate the achievable axial resolution of the system in Fig. 3.

Supplementary Note 5

To observe the cortical and basal sub-structures within the *C. elegans* cuticle we employed the following methodology. The confocal z-plane containing maximum contrast was selected (typically at the mid-height of the specimen) and the cuticle was set as the region of interest by manually identifying the indexes of imaging pixels localised near the cuticle (yellow asterisks in Supplementary Figure 5a-b). Next the spatial gradient between these points was calculated and used to calculate and draw a set of lines that are approximately surface normal to the local position of the cuticle (blue asterisks). The fluorescence intensity values along each individual surface normal were interpolated in 2D to produce a fluorescence profile (blue line in Supplementary Figure 5c). The rising edge of the cuticle was identified (red asterisk in Supplementary Figure 5c) and a window of interest was calculated to ensure that each fluorescence cross-section would contain the same number of values (orange line in Supplementary Figure 5c). This process was repeated for each blue surface normal / cross-section in (a). Each cross-section was normalised such that the initial and maximum values were 0 and 1 respectively. Then the cross-sections were aligned along their x-axes using the positions of the rising edges to ensure that the centre of the cortical cuticle

layer was the same for each cross-section. Then the normalised and aligned cross-sections were averaged to produce a mean cross-section for the specimen (Supplementary Figure 5d). The steps covered by Supplementary Figure 5c-d can be applied to the $\Delta f_B(z)$ cross-sectional measurements (e.g. see Fig. 3g) obtained from phononic imaging for comparison with confocal microscopy (Fig. 4d).

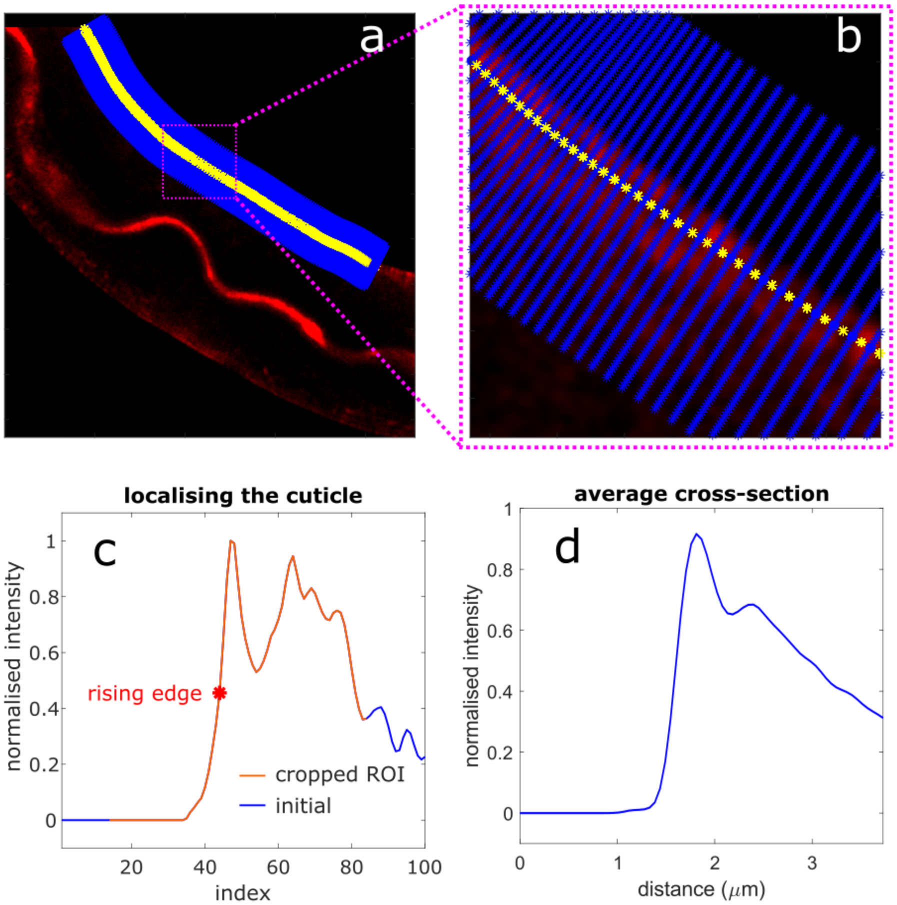

Supplementary Figure 5 a) 2D fluorescence intensity map containing a series of manually-set cuticle markers (yellow asterisks) and calculated surface normals (blue asterisks) as seen in (b). c) The fluorescent intensity profile along each surface normal was extracted and the rising edge (red asterisk) of the cortical layer was localised and used for windowing the data (orange line). The windowed data for each normal cross-section within a specimen were aligned to the rising edge positions along the x-axis and then these were averaged to produce a mean cuticle cross-sectional profile in (d).

Supplementary Note 6

Thermal gradients along the axial direction give rise to small fluctuations in instantaneous frequency in the time-of-flight signal obtained by the PEM system.[20] Consequently, depth-resolved viscoelastic material property measurements will contain depth-dependent variations (error) in addition to variations as a result of the viscoelastic heterogeneity. The contribution of the former can be estimated by measuring the standard deviation of instantaneous frequency of approximately 100 time-of-flight signals (in control medium), which results in the uncertainty versus z plots shown in Supplementary Figure 6. From these it is apparent that the error on the instantaneous frequency increases as a function of depth which can be improved by further averaging the measurements (Supplementary Figure 6b) at the cost of axial resolution and acquisition speed. Furthermore, frequency precision depends on the width of the daughter wavelet used for time-frequency analysis (as discussed in **Axial resolution**). For example, increasing the wavelet window width by a factor of 2.5 (Supplementary Figure 6a,c) improves precision by roughly the same factor, again at the cost of axial resolution. These contributions to error on the instantaneous frequency measurements must be taken into account when determining viscoelastic variation. For example, after calibrating the depth-dependent errors reported in Supplementary Figure 6a, the measurements reported in Fig. 3f contain approximate variations in viscoelasticity on the order of $\Delta f_B = 60 \pm 50$ MHz (control-medial), $\Delta f_B = 300 \pm 30$ MHz (cortical-basal), $\Delta f_B = 600 \pm 40$ MHz (strut), and $\Delta f_B =$ -200$\pm$30 MHz (hypodermal).

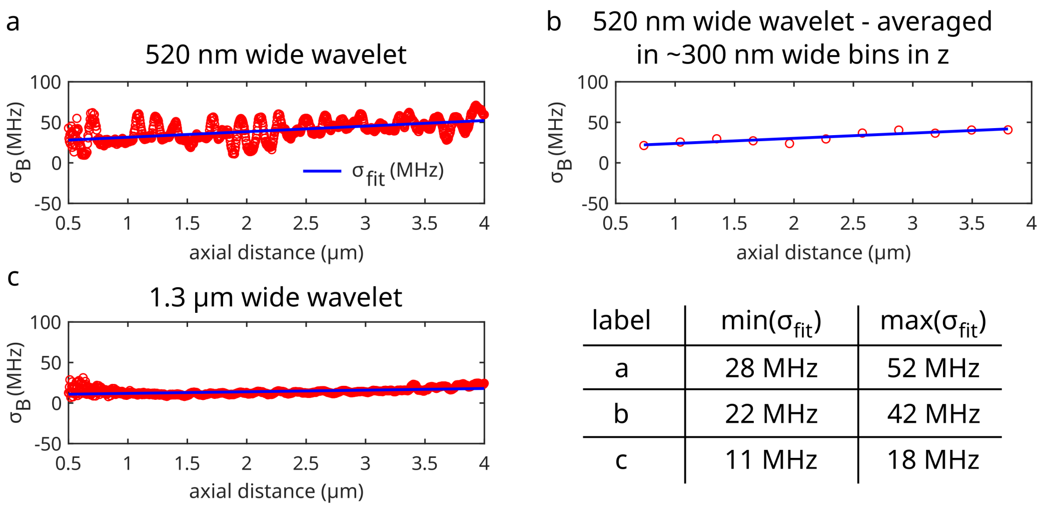

label	min(σ_{fit})	max(σ_{fit})
a	28 MHz	52 MHz
b	22 MHz	42 MHz
c	11 MHz	18 MHz

Supplementary Figure 6 Variation in instantaneous Brillouin frequency shift as a function of depth in the time-of-flight PEM signals. a) Shorter daughter wavelet window widths (Supplementary Figure 4a) result in frequency measurement errors on the order of 28-52 MHz within the depth range 0.5-4 μm. b-d) Further averaging the signal (e.g. into 300 nm spaced bins) improves precision as does increasing the wavelet width at the cost of axial resolution.

Supplementary Note 7

The lateral resolution of the PEM imaging technique is dictated by smaller of the pump and probe beam spot sizes at the core of the distal end of the optical fibre. The pump spot generates a region of coherent acoustic phonons, and the probe beam optically samples the propagation of these phonons. Therefore the overlapped region between the pump and probe volumes will correspond to the maximum Brillouin scattering cross section and therefore the lateral extent of the interaction. Consequently the mode field diameter of the optical fibre at the shorter pump wavelength will determine the lateral resolution. Due to this pump-probe mechanism, and the dependence of the lateral resolution the optics, spatial resolution can be characterised without needing to actually measure the transition in Brillouin frequency between two different materials. Instead, simply the optical intensity can be detected as the fibre-probe is scanned over an edge, e.g. the bar of a US Air Force target. Supplementary Figure 7 presents four scans, two across a vertical edge of the bar (red and blue) and two across a horizontal edge (green and black). Lateral resolution is

approximated as the width of the central transitional region of the edge spread functions, defined by the positions of 20% and 80% amplitude relative to the maximum response ESF = 1 (circular markers in Supplementary Figure 7): r_{psf} = 2.05±0.19 μm.

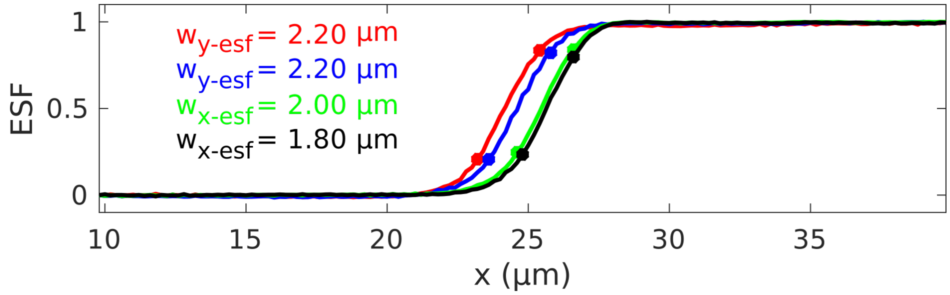

Supplementary Figure 7 Edge spread functions for the PEM system as a result of scanning the fibre-probe across the edge of a resolution target. Two scans were performed in the y-direction and two in the x-direction. The width of each edge response is approximated as the positions of 20% and 80% relative amplitude (circular markers) and the average lateral resolution of the system was determined to be r_{psf} = 2.05±0.19 μm.

Supplementary Note 8

A simple free-space imaging system was constructed opposite the optical fibre portion of the experiment for the purposes of positioning the fibre-probe and providing a point of comparison between optical and phononic imaging. A green LED, condenser, and objective lens (Olympus, LUCPlanFL N, 20x magnification and 0.45 NA) provided Kohler-illumination for reflection mode free-space imaging (Fig. 1a and inset). However, poor optical contrast between medium (PBS) and highly transparent specimens (e.g. mammalian cells in culture) prohibits purely optical localisation Supplementary Figure 8-left). We then introduced a white LED ring to the fibre scanning facility to provide a source of transmission darkfield (DF) illumination whereby diffuse cell-scattered light produces sufficient contrast with the otherwise dark background (Supplementary Figure 8-right)). Both brightfield and darkfield imaging systems provide convenient mechanisms for orienting the fibre-probe with respect to the specimens, but are not strictly needed to perform the final phononic

measurements. Furthermore, their ancillary role in the measurement system can be fulfilled by pre-existing optical endoscopic and endo-microscopy/endocytoscopy imaging technologies.

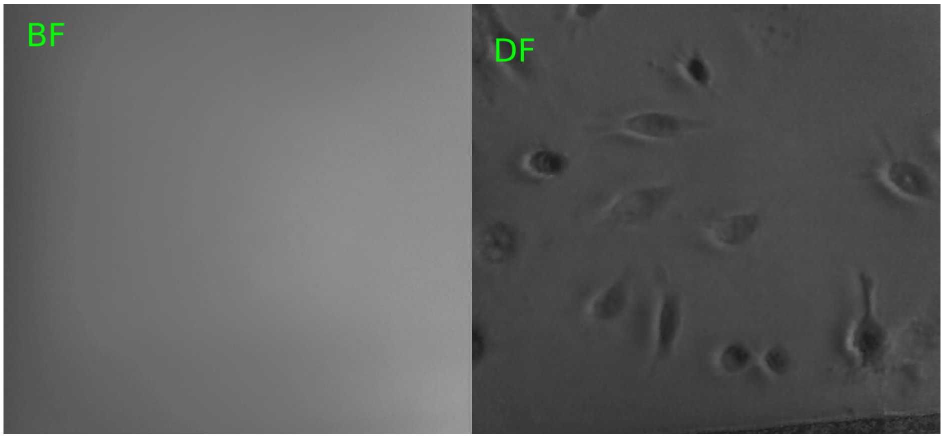

Supplementary Figure 8 left-BF) The intrinsic low contrast performance of the epi-reflection brightfield optical imaging system that supplements the PEM system (specimen is fixed NIH/3T3 fibroblast cells). right-DF) By integrating a custom white-LED ring (diameter is $\approx 3\ cm$) into the fibre-scanning mechanism (fixed between the fibre-clamp and the z-nanopositioner) significantly better optical contrast is achieved (same imaging region as BF). This solution provides oblique transmission illumination through the sample that reduces the contribution of the unscattered DC background light at the imaging sensor (see Fig. 1a for a simplified diagram of this ancillary imaging system, BF and DF).

Supplementary References

44 C. Thomsen, H. T. Grahn, H. J. Maris, *et al.*, "Surface generation and detection of phonons by picosecond light pulses," *Phys. Rev. B* **34**, 4129–4138 (1986).

45 C. Thomsen, J. Strait, Z. Vardeny, *et al.*, "Coherent phonon generation and detection by picosecond light pulses," *Phys. Rev. Lett.* **53**, 989–992 (1984).

46 O. Matsuda and O. B. Wright, "Reflection and transmission of light in multilayers perturbed by picosecond strain pulse propagation," *J. Opt. Soc. Am. B* **19**, 3028–3041 (2002).

47 R. J. Smith, F. Pérez-Cota, L. Marques, *et al.*, "3D phonon microscopy with sub-micron axial-resolution," *Scientific Reports* **11**, 3301 (2021).

48 S. La Cavera, F. Pérez-Cota, R. J. Smith, *et al.*, "Phonon imaging in 3D with a fibre probe," *Light: Science & Applications* **10**, 91 (2021).
