# Supplementary material for: Label-free Brillouin endo-microscopy for the quantitative 3D imaging of sub-micrometre biology
Source: Commun Biol. 2024 Apr 15;7:451. doi: 10.1038/s42003-024-06126-4 (PMC11018753; doi:10.1038/s42003-024-06126-4)
Supplement: Supplementary file 4 — Reporting Summary [file 42003_2024_6126_MOESM4_ESM.pdf]

nature portfolio

Corresponding author(s): Salvatore La Cavera III

Last updated by author(s): 28/2/24

Reporting Summary

Nature Portfolio wishes to improve the reproducibility of the work that we publish. This form provides structure for consistency and transparency in reporting. For further information on Nature Portfolio policies, see our Editorial Policies and the Editorial Policy Checklist.

Statistics

For all statistical analyses, confirm that the following items are present in the figure legend, table legend, main text, or Methods section.

n/a	Confirmed	
☐	☒	The exact sample size (*n*) for each experimental group/condition, given as a discrete number and unit of measurement
☐	☒	A statement on whether measurements were taken from distinct samples or whether the same sample was measured repeatedly
☒	☐	The statistical test(s) used AND whether they are one- or two-sided<br>*Only common tests should be described solely by name; describe more complex techniques in the Methods section.*
☒	☐	A description of all covariates tested
☒	☐	A description of any assumptions or corrections, such as tests of normality and adjustment for multiple comparisons
☐	☒	A full description of the statistical parameters including central tendency (e.g. means) or other basic estimates (e.g. regression coefficient) AND variation (e.g. standard deviation) or associated estimates of uncertainty (e.g. confidence intervals)
☒	☐	For null hypothesis testing, the test statistic (e.g. *F*, *t*, *r*) with confidence intervals, effect sizes, degrees of freedom and *P* value noted<br>*Give P values as exact values whenever suitable.*
☒	☐	For Bayesian analysis, information on the choice of priors and Markov chain Monte Carlo settings
☒	☐	For hierarchical and complex designs, identification of the appropriate level for tests and full reporting of outcomes
☒	☐	Estimates of effect sizes (e.g. Cohen's *d*, Pearson's *r*), indicating how they were calculated

Our web collection on statistics for biologists contains articles on many of the points above.

Software and code

Policy information about availability of computer code

Data collection	Experimental hardware control and data collection were performed using custom in-house developed C/C++ programmes.
Data analysis	Custom MATLAB R2023b (MathWorks) scripts were written for all post-acquisition signal processing and are available upon request.

For manuscripts utilizing custom algorithms or software that are central to the research but not yet described in published literature, software must be made available to editors and reviewers. We strongly encourage code deposition in a community repository (e.g. GitHub). See the Nature Portfolio guidelines for submitting code & software for further information.

Data

Policy information about availability of data

All manuscripts must include a data availability statement. This statement should provide the following information, where applicable:

- Accession codes, unique identifiers, or web links for publicly available datasets
- A description of any restrictions on data availability
- For clinical datasets or third party data, please ensure that the statement adheres to our policy

Experimental data are available upon reasonable request.

Human research participants

Policy information about studies involving human research participants and Sex and Gender in Research.

Reporting on sex and gender	n/a
Population characteristics	n/a
Recruitment	n/a
Ethics oversight	n/a

Note that full information on the approval of the study protocol must also be provided in the manuscript.

Field-specific reporting

Please select the one below that is the best fit for your research. If you are not sure, read the appropriate sections before making your selection.

☒ Life sciences ☐ Behavioural & social sciences ☐ Ecological, evolutionary & environmental sciences

For a reference copy of the document with all sections, see nature.com/documents/nr-reporting-summary-flat.pdf

Life sciences study design

All studies must disclose on these points even when the disclosure is negative.

Sample size	A sample size of n=5 mammalian cells (1 HeLa, 2 3T3 mouse fibroblasts, and 2 MG-63 cells) and n=3 C. elegans larvae were used without sample size calculation. This was to demonstrate proof of concept 3D imaging for our new technology. n=3 C. elegans larvae were imaged using confocal fluorescence microscopy to reveal the existence of cuticle structure.
Data exclusions	Phonon and confocal data that contained motion artefacts.
Replication	The data associated with manuscript Fig. 3 was scanned twice to demonstrate repeatability for different scanning speeds (see Supplement Fig. 7).
Randomization	Phononic imaging ogranisms were selected randomly; confocal imaging organisms were selected to have similar nematode diameter compared with phononic imaging organisms.
Blinding	Blinding not relevant to the imaging proof-of-concept studies performed here.

Reporting for specific materials, systems and methods

We require information from authors about some types of materials, experimental systems and methods used in many studies. Here, indicate whether each material, system or method listed is relevant to your study. If you are not sure if a list item applies to your research, read the appropriate section before selecting a response.

Materials & experimental systems

n/a	Involved in the study	
☒	☐	Antibodies
☐	☒	Eukaryotic cell lines
☒	☐	Palaeontology and archaeology
☐	☒	Animals and other organisms
☒	☐	Clinical data
☒	☐	Dual use research of concern

Methods

n/a	Involved in the study	
☒	☐	ChIP-seq
☒	☐	Flow cytometry
☒	☐	MRI-based neuroimaging

Eukaryotic cell lines

Policy information about cell lines and Sex and Gender in Research

Cell line source(s)	MG-63 (ATCC; CRL-1427), HeLa (ATCC; CCL-2), NIH/3T3 (ATCC; CRL-1658)

Authentication	None of the cell lines used were authenticated
Mycoplasma contamination	All cell lines tested negative for mycoplasma contamination as tested using MycoAlert mycoplasma detection kit (Lonza; LT07-318)
Commonly misidentified lines (See ICLAC register)	n/a

Animals and other research organisms

Policy information about studies involving animals; ARRIVE guidelines recommended for reporting animal research, and Sex and Gender in Research

Laboratory animals	Caenorhabditis elegans N2 wild-type Bristol strain larve nematodes, sourced from the Caenorhabditis Genetics Center (CGC). Nemtodes were cultered feeding on Escherichia coli OP50 strain and nematode growth medium agar at 20 °C.
Wild animals	Not applicable - C. elegans N2 wildtype strain, and Es coli OP50 strain which it feeds on, were sourced from the Caenorhabditis Genetics Center (CGC).
Reporting on sex	C. elegans are hermaphroditic, with a a few that a male sexed in a population. The study primarily focuses on wild-type larvae characteristics without specific reference to sex, collected through life-cycle synchronization and filtration using a Nylon 20 µm filter.
Field-collected samples	Not applicable as C. elegans N2 wild-type Bristol were lab-cultured.
Ethics oversight	The study was conducted following relevant guidelines and regulations for the use of invertebrate research organisms, which currently do not require ethical approval at host organization.

Note that full information on the approval of the study protocol must also be provided in the manuscript.
